# The epidemiological landscape of bloodstream infections in children undergoing chemotherapy or haematopoietic cell transplantation: A retrospective study by Infectious Diseases Working Group of Italian Association of Pediatric Hematology and Oncology (AIEOP)

**DOI:** 10.1111/bjh.70036

**Published:** 2025-09-21

**Authors:** Francesco Baccelli, Francesca Compagno, Gloria Tridello, Francesco Delle Cave, Daniele Zama, Riccardo Masetti, Maria Grazia Petris, Lorenzo Chiusaroli, Manuela Spadea, Francesca Trevisan, Antonella Colombini, Samantha Conci, Cristina Meazza, Paola Muggeo, Maria Vittoria Micheletti, Alessia Pancaldi, Rosamaria Mura, Daniela Onofrillo, Raffaella De Santis, Milena La Spina, Elio Castagnola, Katia Perruccio, Angelica Barone, Nagua Giurici, Angelamaria Petrone, Federico Mercolini, Simona Rinieri, Simone Cesaro, Francesca Carraro7, Annalisa Tondo9, Laura Rachele Bettini10, Elena Pletto2, Olga Nigro11, Roberta Martino12, Eva Parolo13, Giovanni Palazzi12

**Affiliations:** ^1^ Pediatric Hematology and Oncology IRCCS Azienda Ospedaliero‐Universitaria di Bologna Bologna Italy; ^2^ Paediatric Haematology and Oncology Fondazione IRCCS Policlinico San Matteo Pavia Italy; ^3^ Pediatric Hematology Oncology, Department of Mother and Child Azienda Ospedaliera Universitaria Integrata Verona Verona Italy; ^4^ Pediatric Emergency Unit IRCCS Azienda Ospedaliero‐Universitaria di Bologna Bologna Italy; ^5^ Clinic of Pediatric Hemato‐Oncology, Department of Women's and Children's Health, Clinic of Pediatric Hemato‐Oncology University of Padua Padua Italy; ^6^ Division of Pediatric Infectious Diseases, Department for Women's and Children's Health University Hospital of Padua Padua Italy; ^7^ Pediatric Onco‐Hematology, Stem Cell Transplantation and Cellular Therapy Division Regina Margherita Children's Hospital Turin Italy; ^8^ University of Turin Turin Italy; ^9^ Meyer Pediatric Onco‐Hematology Unit‐AOU Meyer Firenze Florence Italy; ^10^ Fondazione IRCCS, Ospedale San Gerardo dei Tintori Monza Italy; ^11^ Pediatric Oncology Unit Fondazione IRCCS Istituto Nazionale dei Tumori Milan Italy; ^12^ Pediatric Oncology‐Hematology Unit, Department of Pediatrics Azienda Ospedaliero Universitaria Policlinico Bari Italy; ^13^ Pediatric Hematology Azienda Ospedaliero Universitaria Pisana Pisa Italy; ^14^ Pediatric Oncology and Hematology Unit, Department of Medical and Surgical Sciences for Mother Children and Adults University of Modena and Reggio Emilia Modena Italy; ^15^ Pediatric Onco‐Hematology Unit Azienda Ospedaliera Brotzu Cagliari Italy; ^16^ Hematology Unit Hospital of Pescara Pescara Italy; ^17^ Pediatric Hematology Oncology Casa Sollievo della Sofferenza Hospital San Giovanni Rotondo Italy; ^18^ Pediatric Hematology and Oncology Unit, AOU Policlinico “Rodolico‐San Marco” University of Catania Catania Italy; ^19^ Infectious Diseases Unit Istituto Giannina Gaslini Children's Hospital Genoa Italy; ^20^ Pediatric Oncology Hematology, Mother and Child Health Department Santa Maria della Misericordia Hospital Perugia Italy; ^21^ Pediatric Onco‐Hematology Unit Azienda Ospedaliero‐Universitaria di Parma Parma Italy; ^22^ Pediatric Hematology‐Oncology Institute for Maternal and Child Health IRCCS “Burlo Garofolo” Trieste Italy; ^23^ U.O.M. Pediatria Ospedale S. Chiara Trento Italy; ^24^ Pediatric Onco‐Hematology Unit, Department of Pediatrics Hospital of Bolzano Bolzano Italy; ^25^ Pediatric Unit, Onco‐Hematology Day Hospital Sant'Anna Hospital Ferrara Italy

**Keywords:** antimicrobial resistance, bloodstream infections, empirical antibiotic therapy, febrile neutropenia, haematopoietic cell transplantation, paediatric haematology, paediatric oncology

## Abstract

Understanding bloodstream infection (BSI) epidemiology is crucial for optimizing antibiotic therapy in paediatric haematology–oncology patients undergoing chemotherapy or haematopoietic cell transplantation (HCT). However, updated paediatric data remain scarce. This multicentre retrospective study analysed BSI epidemiology across 22 Italian centres (2018–2019), assessing pathogens, resistance profiles, empirical antibiotic therapy (EAT) and clinical course with outcomes. Mortality risk factors were evaluated using a Cox regression model. A total of 510 BSI episodes occurred in 396 patients (median age 6.4 years), with an incidence of 2.9 and 5.1 per 1000 inpatient days for chemotherapy and HCT respectively. Multidrug‐resistant (MDR), third to fourth generation cephalosporin‐ and carbapenem‐resistant, account for 18.3%, 29.3% and 8.2% of Gram‐negative infections respectively. 42.2% of *Klebsiella pneumoniae* isolates were MDR. Combination EAT was used in 269/510 episodes, with piperacillin–tazobactam+amikacin being most common. Microbiological appropriateness was 82%. Infection‐related and 30‐day mortality rates were 4.1% and 5.29%, respectively, with appropriate EAT significantly reducing mortality. Our findings highlight the burden of resistant pathogens in paediatric BSIs and emphasize the importance of appropriate EAT in improving outcomes, underscoring the need for treatment strategies tailored to local resistance patterns.

## INTRODUCTION

Bloodstream infections (BSIs) represent a critical complication in patients with cancer or undergoing haematopoietic cell transplantation (HCT), being associated with increased morbidity and mortality.^1^ The aetiology of BSI is mainly bacterial, while fungal pathogens are rare.^2^ Promptly identifying and managing BSI is crucial to improve outcomes. The immediate administration of broad‐spectrum antibiotics is recommended, followed by targeted therapy once pathogens with antibiotic susceptibilities are identified. Current paediatric European guidelines (ECIL‐8) recommend monotherapy with an antipseudomonal non‐carbapenem β‐lactam and β‐lactamase inhibitor, or with fourth‐generation cephalosporin, for clinically stable patients at low risk of resistant infections, while carbapenem monotherapy or combination with a second anti‐Gram‐negative and/or a glycopeptide should be reserved for clinically unstable patients.^3^ A growing concern is the rise of antimicrobial resistance among bacterial pathogens.^4^ The emergence of multidrug‐resistant (MDR) Gram‐negative (GN) bacteria poses significant challenges.^5^ Resistant pathogens limit the effectiveness of standard antibiotics, resulting in delayed appropriate therapy and more severe infections with increased mortality. Local epidemiology of BSI has therefore to be considered in order to correctly guide empiric antibiotic therapy (EAT).^6^, ^7^ EAT needs to be tailored on resistance patterns in colonized patients or in centres with high resistance rates.^3^ Recent European studies addressed the epidemiological trends and management of BSI in adult onco‐haematological patients.^8^, ^9^ Some studies have explored the paediatric setting, but recent data are lacking.^2^, ^10^ Available paediatric data were generally derived from single centres' experiences or mixed reports including also adults.^5^, ^11^ Moreover, the epidemiology of BSI has been extensively described among Italian adult haematology–oncology centres, reporting a dramatic increase in resistant isolates, particularly carbapenem‐resistant bacteria, in recent years.^4^, ^9^, ^12^, ^13^ Comparable paediatric reports, exploring the possible rise in resistance, could be useful in determining the best EAT. This study aims to explore the epidemiology of BSI in the paediatric population among Italian haematology–oncology centres of *Associazione Italiana di Ematologia e Oncologia Pediatrica* (AIEOP) during a recent 2‐year period (2018–2019), examining epidemiological trends, pathogens' profiles with resistance rates, clinical outcomes and appropriateness of EAT.

## METHODS

### Study design

This observational, retrospective, multicentre study was conducted in 22 paediatric haematology and oncology centres of AIEOP. The study was approved by the Ethics Committee of each participating centre. The primary objective was to evaluate the aetiology of BSI in the Italian paediatric haematology–oncology population. Secondary objectives were to assess the antibiotic resistance profiles, the appropriateness of EAT and the clinical course of BSI episodes.

### Data collection

The following inclusion criteria were adopted: (1) age <18 years, (2) diagnosis of febrile BSI (at least one blood culture positive for bacterial and/or fungal growth obtained from a febrile patient) in the period from January 2018 to December 2019, (3) oncological or haematological diagnosis treated with chemotherapy or HCT. Prevalence of BSI was estimated as the number of BSI on the number of diagnoses receiving chemotherapy and the number of HCT per year among the participating centres and on 1000 hospital inpatient days. Single patients' medical records were retrospectively collected using a paper case report form (CRF) by each participating centre. Anonymized data were transferred to the coordinating centre (Pediatric Hematology‐Oncology, Verona) for analysis. EAT was evaluated as appropriate if the microorganism isolated from the blood culture was susceptible to at least one of the antibiotics used empirically in the first 48 h. Pathogens were recorded as susceptible or resistant based on the local microbiology laboratory following EUCAST criteria. MDR strains were defined as germs resistant to three or more antimicrobial classes potentially active, as per the international expert proposal definition.^14^ Specific resistance mechanisms, specifically extended‐spectrum β‐lactamase‐producing Enterobacterales (ESBL‐E) and carbapenem‐resistant Enterobacterales (CRE), were also reported.^15^ BSI episode was defined as ‘severe’ if at least one among paediatric intensive care unit (PICU) admission, respiratory/fluid/aminic/renal support or infection‐related death was reported. Neutropenia was defined as severe when the absolute neutrophil count (ANC) was less than 0.5 × 10^9^/L and very severe when ANC was less than 0.1 × 10^9^/L. The presence of graft‐versus‐host disease (GVHD) was examined in the case of allogenic HCT (allo‐HCT) and graded reporting the worst score in the 7 days before the BSI.

### Statistical analysis

The main characteristics of patients were reported by descriptive statistics. Median, minimum and maximum values were used for continuous variables, while absolute and percentage frequency were used for categorical variables. Comparisons between categorical variables were performed by chi‐squared or Fisher's exact test, as appropriate. The 30‐day, 90‐day and overall mortality were estimated by using the Kaplan–Meier method, considering death due to any cause as an event and the time from infection to the latest follow‐up as survival time; differences between groups were tested by the log‐rank test. Infection‐related mortality was calculated considering death due to BSI episode as an event. Univariate and multivariate risk factor analyses for survival were performed with a Cox regression model. A *p*‐value <0.05 was considered statistically significant. All *p*‐values were two‐sided. All the analyses were performed using the statistical software SASv9.4 (SAS Institute Inc., mCaerdy, iNaCn).

## RESULTS

### Prevalence of BSI

During the study period, 510 BSIs were registered in 396 patients. The prevalence of BSI on the total number of diagnoses receiving chemotherapy (*n* = 1899) among the participating centres was 17.64% and 22.28% on the total number of transplants (*n* = 606). BSI incidence resulted in 2.9/1000 hospital inpatient days for patients receiving chemotherapy and 5.1/1000 days for transplanted patients.

### Patient and BSI characteristics

Median age at BSI onset was 6.4 years (range, 0–18). Two hundred and fourteen (54%) patients were male and 182 (46%) were female. The most frequent underlying conditions were acute lymphoblastic (*n* = 171) and myeloid (*n* = 53) leukaemia (see Supporting Information). Three hundred and eleven (61.0%) episodes occurred in patients during first‐line treatment, 96 (18.8%) during therapy for disease relapse or after autologous HCT (auto‐HCT) and 103 (20.2%) after allo‐HCT. BSI characteristics are listed in Table 1. Hypoxaemia and hypotension were reported in 33 (6.5%) and 40 (7.8%) patients respectively. Severe neutropenia was detected at the onset of BSI in 381 cases (74.7%), being very severe in 330 (64.7%). The median maximum value of C‐reactive protein (CRP) and procalcitonin (PCT) at the onset of BSI was 9.6 mg/dL (range, 0–752) and 2.6 ng/mL (range, 0–412) respectively. Antibacterial prophylaxis or therapy was ongoing in 67 (13.1%) patients at the time of BSI onset, with a higher percentage in allo‐HCT (24.3%) than first diagnosis (11.6%) and relapse/auto‐HCT (6.3%). Radiological findings of pneumonia and pleural effusion were detected in 60 (10.2%) and 8 (1.6%) cases respectively. Among patients who received allo‐HCT, acute GVHD was reported in 15 episodes (12.6%, 15/117), being grade III–IV in 7 (6.0%), while chronic GVHD was observed in 11 episodes (12.2%), extended in 7 (7.8%). Intestinal colonization by resistant organisms was documented by rectal swab in 64 (11.3%) of BSI episodes with no significant difference according to treatment phase (*p* = 0.8). Colonizing bacteria included 26 ESBL‐producing bacteria, 23 carbapenemase‐producing bacteria, 3 both ESBL and carbapenemase‐producing bacteria and 12 other resistant microorganisms.

**TABLE 1:** BSI episodes’ clinical characteristics.

	BSI episodes (*n* = 510)	First diagnosis (*n* = 311)	Relapse and auto‐HCT (*n* = 96)	Allo‐HCT (*n* = 103)
Fever°C—median (range)	38.6 (37–40.8)	38.7 (37.0–40.8)	38.6 (37.7–40.3)	38.5 (37.5–40.3)
Fever >39°C—no. (%)	187 (36.7)	123 (39.5)	38 (39.6)	26 (25.2)
Hypoxaemia (SpO2 < 92%)—no. (%)	33 (6.5)	17 (5.5)	3 (3.1)	13 (12.6)
Hypotension (SBP < 90 mmHg)—no. (%)	40 (7.8)	19 (6.1)	7 (7.3)	14 (13.6)
Seizures—no. (%)	4 (0.8)	2 (0.6)	1 (1.0)	1 (1.0)
Presence of CVC—no. (%)	499 (97.8)	303 (97.4)	96 (100.0)	100 (97.1)
Presence of urinary catheter—no. (%)	14 (2.7)	8 (2.6)	4 (4.2)	2 (1.9)
Severe neutropenia (<500/mmc)—no. (%)	381 (74.7)	244 (78.5)	70 (72.9)	67 (65.0)
Profound neutropenia (<100/mmc)—no. (%)	330 (64.7)	207 (66.6)	62 (64.6)	61 (59.2)
Neutropenia duration—days—median (range)	12 (1–398)	10 (1–71)	14 (1–101)	21 (1–398)
Lymphocyte count (n/mmc)—median (range)	200 (0–2900)	240 (0–2900)	200.0 (0. 2620)	0.0 (0–1588)
Steroid therapy in the previous 7 days—no. (%)	152 (29.8)	95 (30.5)	28 (29.2)	29 (28.2)
Dose of steroid therapy (median mg/kg/day)	2 (0–93)	2.1 (0–93)	1.9 (0.2–6.0)	1 (0.1–6.7)
Antibacterial prophylaxis (or antibacterial therapy in the previous 7 days)—no. (%)	67 (13.1)	36 (11.6)	6 (6.3)	25 (24.3)
Antifungal prophylaxis—no (%)	232 (45.5)	93 (29.9)	55 (57.3)	84 (81.6)
Antiviral prophylaxis—no. (%)	148 (29.0)	26 (8.4)	35 (36.5)	87 (84.5)
CRP—mg/dL—median (range)	9.6 (0–752.0)	9.9 (0.0–752.0)	10.8 (0.5–41.7)	8.9 (0.0–130.0)
PCT—ng/mL—median (range)	2.6 (0–412)	2.6 (0.0–412)	3.8 (0–184)	1.5 (0.0–85)
Pulmonary radiological findings—no. (%)				
Pneumonia with/without pleural effusion	60 (10.2)	29 (9.4)	11 (11.5)	20 (19.5)
Pleural effusion	8 (1.6)	3 (1.0)	1 (1.0)	4 (3.9)
Acute GVHD (allo‐HCT pts)—no. (%)				
Grade I–II				8 (6.8)
Grade III–IV				7 (6)
Chronic GVHD (allo‐HCT pts alive at day 100)—no. (%)				
Limited				4 (4.4)
Extensive				7 (7.8)
Organ toxicity—no. (%)				
Grade I–II	192 (37.6)	116 (37.3)	40 (41.7)	36 (35.0)
Grade III	89 (17.5)	44 (14.1)	25 (26.0)	20 (19.4)
Grade IV	33 (6.5)	15 (4.8)	3 (3.1)	15 (14.6)
Intestinal colonization—no. (%)	57 (11.2)	33 (10.6)	12 (12.5)	12 (11.7)
ESBL	24 (4.7)	11 (3.5)	8 (8.3)	5 (4.9)
CPE	20 (3.9)	16 (5.1)	2 (2.1)	2 (1.9)
ESBL + CPE	3 (0.6)	0 (0.0)	0 (0.0)	3 (2.9)
Other	10 (2.0)	6 (1.9)	2 (2.1)	2 (1.9)

Abbreviations: allo, allogenic; auto, autologous; BSI, bloodstream infection; CRP, C‐reactive protein; CVC, central venous cathether; GVHD, graft‐versus‐host disease; HCT, haematopoietic cell transplantation; IFI, invasive fungal infection; PCT, procalcitonin; SBP, systolic blood pressure.

### Aetiology and resistance

During 510 BSI, 527 positive blood cultures were documented. In 36 cases, ≥1 microorganism (two in 35 and three in 1 case) was found. Overall, 564 pathogens were detected (see Table 2), including 239 Gram‐positive (GP) bacterial (46.9%), 242 GN bacterial (47.5%), 3 fungal (0.6%), 1 mycobacterial (0.2%) and 25 polymicrobial mixed (4.9%) BSIs. Polymicrobial infections included 24 episodes caused by both GP and GN bacteria and 1 caused by GN and fungal organisms. No differences in the percentage of GP versus GN isolates were reported according to treatment phase (*p* = 0.9). The most frequent GP isolates were staphylococci (61.1%) (*S. aureus* 11.8%; *coagulase‐negative staphylococci*—CONS 49.3%) followed by streptococci (17.9%) and enterococci (7.1%). Among GN, 186 (66.7%) were Enterobacteriaceae, being *E*. *coli* the most frequent GN isolate (33%), followed by *K*. *pneumoniae* (17.6%) (*K.Pn*) and *P*. *aeruginosa* (17.2%) (*Ps.A*). No significant difference in the percentage of *Ps.A*, *E. coli* and *K.Pn* was documented according to treatment phase (*p* = 0.5). As shown in Table 3, three of 33 (9.1%) *S. aureus* isolates were methicillin‐resistant (MRSA) without any resistance to vancomycin. Vancomycin resistance occurred in three (15%) enterococci strains (VRE). Among GN bacteria, 45 (16.1%) MDR strains were reported, as shown in Table 4, with no significant difference in MDR percentage according to treatment phase (*p* = 0.3). MDR prevalence was higher among *K.Pn* (42.2%). An overall resistance rate of 29.4%, 22.9%, 17.6%, 8.2% and 29.7% to III–IV generation cephalosporins, piperacillin–tazobactam, aminoglycosides, carbapenems and quinolones, respectively, was reported. Resistance to carbapenems was reported in 14.3% and 16.7% of *K.Pn* and *Ps.A* strains respectively. Five isolates (1.8%) were resistant to ceftazidime–avibactam and six (2.2%) to ceftolozane/tazobactam, including three *K.Pn* strains resistant to both combinations. *K.Pn* isolates were also highly resistant to cephalosporins (57.1%) and piperacillin–tazobactam (51%) compared to other strains. Among Enterobacteriaceae, 23.7% isolates (44/144) were ESBL‐producing, and 9% (13/144) carbapenemase‐producing, with the highest resistance in *K.Pn* (42.9% ESBL‐producing and 14.3% carbapenemase‐producing). Nineteen of 92 *E. coli*‐BSI (20.7%) were caused by ESBL‐producing strains. In colonized patients, 28.6% developed *E. coli* BSI (6/21), compared to 18.3% in patients without known colonization (13/71). *K.Pn* and *Enterobacter* spp. BSI were caused by carbapenemase‐producing strains in 8 of 49 cases (16.3%) and 4 of 30 cases (13.3%), respectively, with a higher percentage in colonized (*K.Pn*: 53.8%, 7/13; *Enterobacter*: 33.3%, 1/3) versus non‐colonized (*K.Pn*: 2.8%, 1/36; *Enterobacter*: 7.4%, 2/27) patients (see Supporting Information).

**TABLE 2:** Microorganisms isolated among the 527 positive blood cultures.

	Isolated microorganisms (*n* = 564)	First diagnosis (*n* = 335)	Relapse and auto‐HCT (*n* = 109)	Allo‐HCT (*n* = 120)
Gram‐positive bacteria—no. (%)	**280 (49.6)**	**166 (49.6)**	**52 (47.7)**	**62 (51.7)**
*Staphylococcus aureus*	33 (11.8)	26 (15.7)	1 (1.9)	6 (9.7)
CONS	138 (49.3)	82 (49.4)	28 (53.8)	28 (45.2)
*Streptococcus mitis/oralis*	34 (12.1)	17 (10.2)	4 (7.7)	13 (21.0)
*Streptococcus pneumoniae*	6 (2.2)	3 (1.8)	2 (3.8)	1 (1.6)
Other streptococci (non‐mitis/oralis, non‐pneumoniae)	10 (3.6)	5 (3.0)	4 (7.7)	1 (1.6)
*Enterococcus faecium/faecalis*	19 (6.9)	6 (3.6)	5 (9.6)	8 (12.9)
Other enterococci (non‐faecium/faecalis)	1 (0.3)	0 (0.0)	1 (1.9)	0 (0.0)
Other Gram‐positive bacteria	39 (13.9)	27 (16.3)	7 (13.5)	5 (8.1)
Gram‐negative bacteria—no. (%)	**279 (49.5)**	**165 (49.3)**	**56 (51.4)**	**58 (48.3)**
*Escherichia coli*	92 (33.0)	59 (35.8)	16 (28.6)	17 (29.3)
*Klebsiella penumoniae*	49 (17.6)	26 (15.8)	10 (17.9)	13 (22.4)
*Enterobacter cloacae*	27 (9.7)	15 (9.1)	4 (7.1)	8 (13.8)
Other enterobacteriaceae	18 (6.4)	9 (5.5)	7 (12.5)	2 (3.4)
*Pseudomonas Aeruginosa*	48 (17.2)	32 (19.4)	7 (12.5)	9 (15.5)
*Stenotrophomonas maltophila*	6 (2.1)	4 (2.4)	1 (1.8)	1 (1.7)
Other non‐fermenting bacteria	10 (3.6)	4 (2.4)	3 (5.4)	3 (5.2)
Other Gram‐negative bacteria	29 (10.4)	16 (9.7)	8 (14.3)	5 (8.6)
Mycobacteria—no. (%)	**1 (0.2)**	**1 (0.3)**	**0**	**0**
Fungi—no. (%)	**4 (0.7)**	**3 (0.9)**	**1 (0.9)**	**0**
*Candida albicans*	1	1	0	0
Other candida spp.	3	2	1	0

*Note*: Bold values indicate the main categories (gram pos, gram neg, mycobacteria, fungi) in order to highlight them.

Abbreviations: allo, allogenic; auto, autologous; CONS, coagulase‐negative staphylococci; HCT, haematopoietic cell transplantation.

**TABLE 3:** Antibiotic resistance of GP‐isolated bacteria.

	Methicillin, oxacillin	Ampicillin, penicillin	Vancomycin	Linezolid	Daptomycin	Clindamycin
GP bacteria, no (%)						
Streptococci	—	20 (40)	1 (2)	—	—	—
S. Aureus	3 (9.1)	—	0 (0)	0 (0)	1 (3)	—
CONS	99 (71.7)	—	0 (0)	2 (1.4)	3 (2.2)	—
Enterococci	—	15 (75)	3 (15)	—	—	—
Other GP	2 (5.1)	14 (35.9)	1 (2.6)	0 (0)	0 (0)	7 (17.9)
Total GP	104 (37.1)	49 (17.5)	5 (1.8)	2 (0.7)	4 (1.4)	7 (2.5)

Abbreviations: CONS, coagulase‐negative staphylococci; GP, Gram positive.

**TABLE 4:** Antibiotic resistance of GN‐isolated bacteria.

	III–IV gen. cephalosporin	Piperacillin–tazobactam	Aminoglycosides	Carbapenems	Quinolones	Ceftazidime–avibactam	Ceftolozane–tazobactam	MDR
GN bacteria, no. (%)								
*E. coli*	23 (25.0)	16 (17.4)	18 (19.6)	2 (2.2)	34 (37.0)	1 (1.1)	0	11 (12.0)
*K. pneumoniae*	28 (57.1)	25 (51.0)	15 (30.6)	7 (14.3)	23 (46.9)	3 (6.1)	3 (6.1)	19 (42.2)
*P. aeruginosa*	9 (18.8)	6 (12.5)	4 (8.3)	8 (16.7)	12 (25.0)	0	2 (4.2)	4 (8.5)
Other enterobacteriaceae	14 (31.1)	12 (26.7)	7 (15.6)	2 (4.4)	8 (17.8)	0	0	7 (15.6)
*Stenotrophomonas maltophila*	1 (16.7)	0	0	0	0	—	—	0
Oher non‐fermenting bacteria	2 (20.0)	0	0	2 (20.0)	0	—	—	0
Other Gram‐negative	5 (17.2)	5 (17.2)	5 (17.2)	2 (6.9)	6 (20.7)	1 (3.4)	1 (3.4)	4 (50.0)
Total GN	82 (29.4)	64 (22.9)	49 (17.6)	23 (8.2)	83 (29.7)	5 (1.8)	6 (2.2)	45 (18.3)

Abbreviations: GP, Gram positive; MDR, Multidrug resistant.

### EAT and response

Empirical monotherapy or combination of two or more antibacterial agents was adopted in 231 and 269 cases respectively. EAT included cephalosporins in 263 cases (ceftazidime 184, ceftriaxone 49, cefepime 27 and cefotaxime 3), while piperacillin–tazobactam was adopted in 169 cases and meropenem in 48. Two and one patients received ciprofloxacin and amoxicillin‐clavulanic acid as monotherapy respectively. In 207 episodes, an aminoglycoside was added in the first empiric therapy, including amikacin in 201 and gentamicin in 6 cases respectively. An anti‐GP drug was added in 119 cases (teicoplanin in 69, vancomycin in 41, daptomycin in 6 and linezolid in 3). Other agents included in EAT were metronidazole in 15 and levofloxacin in 2 cases respectively. Polyclonal IgM‐enriched immunoglobulins (Pentaglobin®)^16^ were administered empirically at the onset of fever in two cases. Antifungal therapy was used in EAT in 35 cases (liposomal B amphotericin in 12, fluconazole in 11, voriconazole in 8, micafungin in 2, posaconazole in 1 and caspofungin in 1). In 418 cases, the isolated germ was found sensitive to at least one agent used as EAT, resulting in an overall microbiological appropriateness of 82.0%. No significant difference in appropriateness was detected among different treatment phases (*p* = 0.8) (see Supporting Information). As shown in Table 5, clinical complete resolution at 30 and 90 days was observed in 85.5% and 91.9% of cases respectively. Significantly lower rates of complete clinical resolution at 30 days were reported in relapse/auto‐HCT and allo‐HCT compared to first diagnosis (85.9 and 85.3% vs. 93.4; *p* = 0.03). One hundred and forty‐six (28.6%) episodes were defined as ‘severe’, requiring PICU admission in 38 (7.5%), respiratory support in 34 (6.7%), fluid support in 126 (24.7%), vasopressor support in 43 (8.4%) and renal support in 13 (2.5%). Death due to infection was reported in 21 cases (4.1%). A significant difference in the percentage of severe episodes was reported in different treatment phases, representing 47.6% of BSI in allo‐HCT, 35.4% in relapse/auto‐HCT and 20.3% in first diagnosis (*p* < 0.0001). A higher frequency of severe episodes in GN BSI compared to GP BSI was also noted (GN 33% vs. GP 23.8%, *p* = 0.02). No difference regarding clinical resolution and severity was documented based on MDR isolate (see Supporting Information).

**TABLE 5:** The 30‐ and 90‐day outcome and clinical severity of BSI episodes according to aetiology (GP vs. GN), treatment phase or presence of MDR isolate.

	Aetiology			Treatment phase				MDR (for GN only)			
	GP	GN + mixed		First diagnosis	Relapse and auto HCT	Allo HCT		No MDR	MDR		Total
**30‐day outcome**	***N* = 222**	***N* = 213**		***N* = 292**	***N* = 79**	***N* = 92**		***N* = 174**	***N* = 35**		***N* = 463** ^a^
Complete resolution	189 (90.4)	205 (91.1)	0.8	269 (93.4)	66 (85.7)	61 (85.9)	**0.03**	148 (90.8)	30 (90.9)	1	396 (85.5)
Clinical stability or worsening	20 (9.6)	20 (8.9)		19 (6.6)	11 (14.3)	10 (14.1)		15 (9.2)	3 (9.1)		40 (8.6)
*Missing data*	13	13		4	2	21		11	2		27 (5.8)
**90‐day outcome**	***N* = 186**	***N* = 205**		***N* = 249**	***N* = 62**	***N* = 82**		***N* = 155**	***N* = 27**		***N* = 393** ^b^
Complete resolution	171 (96.6)	188 (97.4)	0.7	241 (97.6)	58 (96.7)	62 (95.4)	0.6	141 (97.2)	24 (96.0)	0.6	361 (91.9)
Clinical stability or worsening	6 (3.4)	5 (2.6)		6 (2.4)	2 (3.3)	3 (4.6)		4 (2.8)	1 (4.0)		11 (2.8)
*Missing data*	9	12		2	2	17		10	2		21 (5.3)
**Clinical severity of infection**	***N* = 239**	***N* = 267**		***N* = 311**	***N* = 96**	***N* = 103**		***N* = 191**	***N* = 42**		*N* = 510
Severe infection^c^	57 (23.8)	88 (33.0)	**0.02**	63 (20.3)	34 (35.4)	49 (47.6)	**<0.0001**	61 (31.9)	14 (33.3)	0.9	146 (28.6)
Non‐severe infection	182 (76.2)	179 (67.0)		248 (79.7)	62 (64.6)	54 (52.4)	130 (68.1)	28 (66.7)		364 (71.4)	130 (68.1)

*Note*: Bold values represents the the presence of sub‐categories with spaces at the beginning of the rows. Main categories are “30‐day outcome”, “90‐day outcome” and “clinical severity of infection”.

Abbreviations: allo, allogenic; auto, autologous; BSI, bloodstream infection; GP, Gram‐positive; GN, Gram‐negative; HCT, haematopoietic cell transplantation; MDR, multidrug resistant; PICU, paediatric intensive care unit.

^a^ 47 cases excluded: dead before 30 days.

^b^ 117 cases excluded: dead before 90 days.

^c^ Composite outcome of severe infection: at least one among PICU admission, respiratory support, fluid support, aminic support, renal support or death due to infection.

### Mortality

**FIGURE 1:**
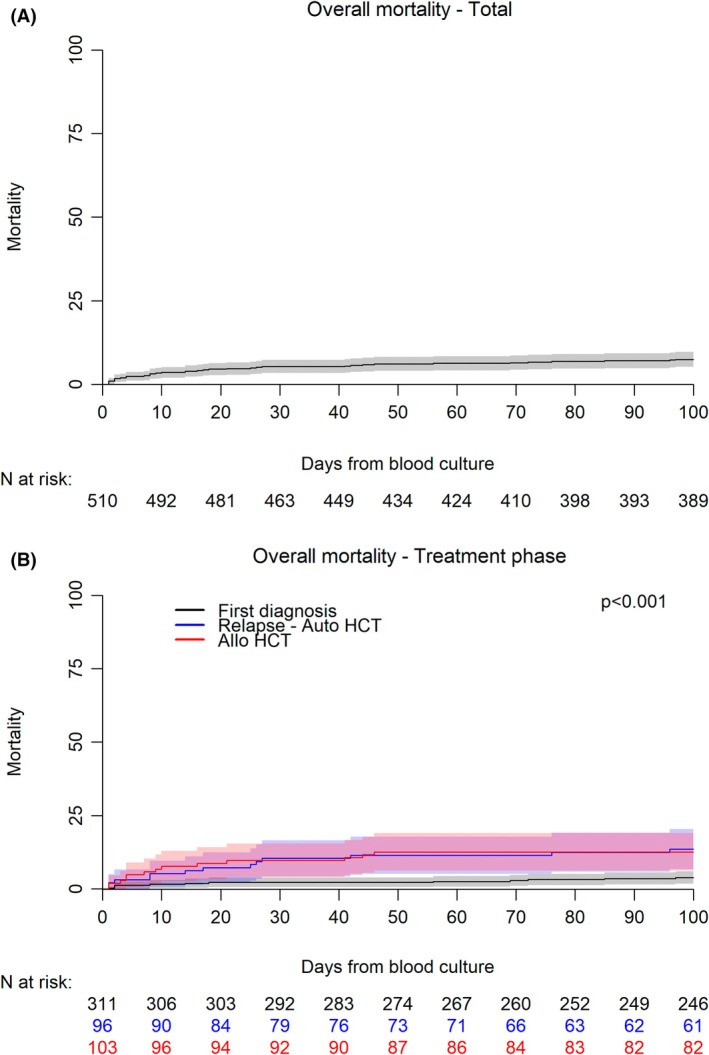
Overall mortality in (A) whole cohort and (B) according to treatment phase. T1, 30 days; T2, 90 days. allo, allogenic; auto, autologous; HCT, haematopoietic cell transplantation

After a median follow‐up of 1.62 years (95% confidence interval, 95% CI: 1.49–1.74), 59 patients were dead at last follow‐up, with an overall mortality rate of 12.18% (95% CI: 9.40–15.34), as shown in Figure 1. The cumulative incidence (CI) of death at 30 and 90 days was 5.29% (95% CI: 3.58–7.48) and 7.06% (95% CI: 5.05–9.50) respectively. Causes of death included 21 infection‐related (see Supporting Information), 36 disease progression and one toxicity (one missing). Estimated infection‐related mortality was 4.13% (95% CI: 2.64–6.12). Factors potentially associated with 30‐day and infection‐related mortality are shown in Table 6. Regarding 30‐day mortality, allo‐HCT, procalcitonin value, septic shock, EAT appropriateness, GN‐BSI and carbapenem‐resistant BSI resulted significantly associated with the outcome in univariate analysis. Allo‐HCT and inappropriateness were confirmed as risk factors in multivariate analysis. Regarding infection‐related mortality, allo‐HCT, procalcitonin value, septic shock, ongoing immunosuppressive therapy, EAT appropriateness, GN‐BSI and carbapenem‐resistant BSI were associated with outcome in univariate analysis and inappropriateness was the only confirmed risk factor in multivariate analysis.

**TABLE 6:** Univariate and multivariate analysis of factors associated with 30‐day mortality and infection‐related mortality.

Risk factors	30‐day mortality				Infection‐related mortality			
	Univariate		Multivariate		Univariate		Multivariate	
	HR (95% CI)	*p*	HR (95% CI)	*p*	HR (95% CI)	*p*		
Sex, F versus M	0.65 (0.30–1.42)	0.3			0.68 (0.28–1.65)	0.4		
Age, continuous	1.02 (0.95–1.09)	0.6			1.05 (0.97–1.13)	0.2		
Treatment phase, allo‐HCT versus first diagnosis or relapse/auto‐HCT	2.39 (1.09–5.22)	**0.03**	3.01 (1.12–8.08)	**0.03**	2.48 (1.03–5.98)	**0.04**		
Fever ≥ 39°C	0.67 (0.29–1.53)	0.3			0.79 (0.32–1.97)	0.6		
CRP (mg/dL) 10‐point effect	1.02 (0.97–1.08)	0.5			1.02 (0.96–1.08)	0.5		
PCT (ng/mL) 10‐point effect	1.13 (1.06–1.20)	**0.0002**			1.13 (1.06–1.20)	**0.0001**		
Hypotension/septic shock	6.52 (2.93–14.53)	**<0.0001**			8.35 (3.45–20.20)	**<0.0001**		
Intestinal colonization	0.99 (0.30–3.28)	1			0.85 (0.20–3.66)	0.8		
ANC < 100/mmc	0.80 (0.37–1.72)	0.6			1.76 (0.65–4.81)	0.3		
Lymphocyte count 10‐point effect	0.98 (0.91–1.07)	0.7			0.94 (0.84–1.06)	0.3		
Immunosuppressive therapy	0.73 (0.28–1.93)	0.5			1.38 (1.08–1.77)	**0.01**		
Steroid therapy	1.38 (0.63–3.02)	0.4			1.18 (0.48–2.93)	0.7		
Appropriateness of empiric antibiotic therapy	3.44 (1.19–9.89)	**0.02**	3.33 (1.16–9.57)	**0.03**	3.96 (1.19–13.16)	**0.02**	3.96 (1.19–13.16)	**0.02**
aetiology, GN versus GP	2.40 (1.05–5.47)	**0.038**			2.18 (0.83–5.74)	0.1		
MDR (GN only)	2.34 (0.80–6.84)	0.12			1.56 (0.42–5.77)	0.5		
Carbapenem‐resistant (GN only)	5.11 (1.57–16.60)	**0.007**			4.91 (1.27–18.98)	**0.02**		
First‐line drug (as EAT) (ceftazidime, piperacillin–tazobactam, meropenem)	1.85 (0.85–4.05)	0.12			1.77 (0.74–4.28)	0.2		
Monotherapy versus combination therapy	0.68 (0.32–1.46)	0.3			0.94 (0.40–2.21)	0.9		
Amynoglycoside added in EAT	0.59 (0.26–1.35)	0.2			0.56 (0.22–1.44)	0.2		
Anti‐GP added in EAT	0.40 (0.12–1.31)	0.13			1.27 (0.49–3.28)	0.6		

Abbreviations: allo, allogenic; ANC, absolute neutrophil count; auto, autologous; CI, confidence interval; CRP, C‐reactive protein; EAT, empiric antibiotic therapy; GN, Gram‐negative; GP, Gram‐positive; HCT, haematopoietic cell transplantation; HR, hazard ratio; MDR, multidrug resistant; PCT, procalcitonin.

## DISCUSSION

We reported a multicentric analysis of BSI in paediatric patients receiving anticancer therapy or undergoing HCT. To our knowledge, this is the first ‘paediatric‐only’ national European report after the publication of ECIL‐4 guidelines in 2013 that recommended escalation therapy in febrile neutropenia (FN) and selected situations in which a de‐escalation approach should be preferred, in order to avoid unnecessary and early use of broader spectrum antibiotics, mainly carbapenems and combinations.^17^ This study refers to BSI developed in Italy in the period 2018–2019, immediately before the publication of paediatric ECIL guidelines, providing information about epidemiological trends and resistance rates in a high‐resistance country.^2^, ^3^

BSI incidence was 2.9/1000 hospital inpatient days for patients receiving chemotherapy and higher in HCT setting (5.1/1000 hospital inpatient days), as expected. An approximately equal prevalence of GN‐ and GP‐BSI was reported, with a more severe clinical course associated with GN aetiology. These findings were similar to other reports by Italian adult consortia.^13^ Intestinal colonization by resistant strains was present in 11% of episodes, including mainly ESBL‐producing and carbapenem‐resistant Enterobacteriaceae (CPE). A recent global meta‐analysis on adult and paediatric patients with haematological malignancies reported higher colonization rates for both ESBL (19%) and CPE (21%). Our data are in line with previous Italian studies on mixed adult and paediatric haematological population showing similar colonization by ESBL (9%) and CPE (2%).^9^, ^18^, ^19^

We reported a higher risk of infection by resistant strains in colonized patients compared to non‐colonized, regarding both ESBL *E. coli* and carbapenem‐resistant *K.Pn* and *Enterobacter*, confirming previous adult studies.^20^, ^21^ Inappropriate empiric therapy has been associated with increased mortality, particularly in GN BSI.^22^ These data strengthen the importance of using EAT active against colonizing bacteria in colonized patients. Further data on current epidemiology rates would be clinically relevant, particularly investigating CPE rates and the role for novel antibiotics in the era of growing resistances.^23^, ^24^ Of note, despite an infrequent use of these drugs in our study, we reported 1.8% and 2.2% resistance to ceftazidime–avibactam and ceftolozane–tazobactam respectively. With the increasing use of these antibiotics as targeted and empirical therapy, attentive surveillance of resistances is mandatory.

Regarding GN‐BSI, there was no difference in the prevalence of MDR or intestinal colonization according to treatment phase, also considering the heavily pretreated cohort of allo‐HCT recipients that generally presented multiple infectious episodes. This may reflect a prominent role of local epidemiology in resistant colonization and infections that needs to be considered in EAT choice.^25^ Future studies will hopefully address the infectious risk among specific subgroups of patients receiving novel therapies, being increasingly used in the paediatric setting.^26^, ^27^, ^28^ We showed a relevant percentage of isolates resistant to both piperacillin/tazobactam (22.9%) and cephalosporins (29.4%), which are the first‐line recommended monotherapies in clinically stable children.^3^ At the same time, a considerable 17.6% of aminoglycoside‐resistant GN were reported, reflecting an epidemiological setting in which nearly half of patients received combination including an aminoglycoside. Exploring current epidemiological trends of resistant patterns, after the implementation of ECIL‐8 guidelines in 2020, can help clinicians in the choice of best EAT to cover GN resistances. Importantly, knowledge of local epidemiology is crucial for the selection of patients who can benefit from aminoglycoside‐containing EAT. Combination proved to be effective in reducing mortality in case of GN bacteraemia, highlighting the importance of developing tools that can help predict GN bacteraemia at the onset of FN.^29^ PCT levels were associated with infection‐related mortality in univariate analysis in our report, suggesting testing it in a prospective manner, together with other novel inflammatory biomarkers and promising microbiome‐derived parameters.^30^, ^31^, ^32^, ^33^

High rates of quinolone‐resistant BSI were reported, generally low compared to adult studies, probably reflecting a lower use of quinolone prophylaxis in paediatric patients in line with recommendations against the indiscriminate use of prophylaxis in children.^34^, ^35^ This recommendation has been highlighted in recent guidelines, and more recent studies will address the trends of quinolone resistance after some years of policy changes.^3^, ^36^, ^37^ Quinolone prophylaxis has demonstrated to be effective in reducing the incidence of bacteraemia in children receiving intensive therapy for acute leukaemia in a recent meta‐analysis, but no impact on survival has been demonstrated.^38^ Open questions in the paediatric setting are still present, particularly how to effectively select patients who may benefit from prophylaxis.

Carbapenem‐resistant BSI account for 8.2% of all episodes. This percentage was higher for *K.Pn*, that also shows a higher MDR percentage than the whole cohort. These data are consistent with adult Italian reports showing a dramatic increase in carbapenem‐resistant *K.Pn* in patients with haematological malignancies.^12^ A relevant percentage of carbapenem‐resistant *Ps.A* was also reported. This trend has also been reported in immunocompromised patients in studies from other European countries.^8^, ^20^, ^39^ Carbapenem‐resistant BSI was associated with mortality in univariate analysis, highlighting the need for continuous epidemiological surveillance and colonization screening and for cautious adoption of carbapenems in haematological patients.

Half of patients received a monotherapy EAT with cephalosporins or piperacillin–tazobactam, while in almost the rest of episodes, empirical therapy included a carbapenem or an aminoglycoside‐containing combination. This latter approach should be reserved for clinically unstable patients or in the presence of a high risk of resistant bacteraemia according to current guidelines. With this approach, we have reported an infection‐related mortality of 4.1% and positive rates of clinical resolution at 30 and 90 days. The percentage of clinically severe infections, including invasive support and PICU admission, was inferior to 30% of episodes.^40^ These generally favourable results raise the question about the use of combinations or carbapenems as EAT for FN, in selected high‐risk patients, particularly in the presence of an unfavourable epidemiology, also considering that EAT microbiological appropriateness resulted in the strongest predictor of infection‐related mortality.

This study is limited by its retrospective design and prospective data on paediatric BSI are certainly needed in order to characterize epidemiological trends in children with onco‐haematological diseases more properly. Selected data on different subgroups of patients, particularly solid versus haematological malignancies, and transplanted patients could add important information. Comprehensive data on FN episodes would be of great interest in order to explore the rate of bacteraemia in these patients, similar to adult counterpart reports, and to determine the impact of BSI on patients' outcomes. More detailed data on colonization would also help in better defining the best EAT approach in colonized patients, including results of respiratory specimens and more detailed microbiome data.

## AUTHOR CONTRIBUTIONS

F.B. and S.C. designed the research study; F.B., F.C. and D.Z. performed the research and wrote the paper; G.T. analysed the data. Other authors performed the research; S.C. supervised and reviewed the paper.

## FUNDING INFORMATION

The work reported in this publication was funded by the Italian Ministry of Health, RC‐2025‐2791682.

## CONFLICT OF INTEREST STATEMENT

The authors declare no conflicts of interest related to this work.

## ETHICS STATEMENT

This study was approved by the Ethics Committee of each participating institution. All procedures were conducted in accordance with the Declaration of Helsinki.

## Supporting information


Data S1.

## Data Availability

The data of this study are available at 10.5281/zenodo.17132274.
