# Supplementary material for: The epidemiological landscape of bloodstream infections in children undergoing chemotherapy or haematopoietic cell transplantation: A retrospective study by Infectious Diseases Working Group of Italian Association of Pediatric Hematology and Oncology (AIEOP)
Source: Br J Haematol. 2025 Sep 21;207(5):1982–93. doi: 10.1111/bjh.70036 (PMC12624170; doi:10.1111/bjh.70036)
Supplement: Supplementary file 1 — Data S1. [file BJH-207-1982-s001.docx]

**Supplementary material**

**Supplementary Table S1. Patients baseline characteristics; CNS, central nervous system; HCT, hematopoietic cell transplantation**

|  | **Patients (n = 396)** |
| --- | --- |
| **Age at diagnosis – median (range)** | 6.4 (0-18) |
| **Gender – no. (%)** |  |
| **Male** | 214 (54.0) |
| **Female** | 182 (46.0) |
| **Diagnosis – no. (%)** |  |
| **Acute lymphoblastic leukemia** | 171 (43.2) |
| **Acute myeloid leukemia** | 53 (13.4) |
| **Myelodysplastic syndrome** | 7 (1.8) |
| **Lymphoma** | 35 (8.8) |
| **Bone marrow failure syndromes** | 14 (3.5) |
| **Other benign diseases (undergoing HCT)** | 29 (7.3) |
| **CNS tumors** | 23 (5.8) |
| **Neuroblastoma** | 18 (4.5) |
| **Chronic myeloid leukemia** | 2 (0.5) |
| **Wilms tumor** | 5 (1.3) |
| **Ewing sarcoma** | 12 (3.0) |
| **Rabdomyosarcoma** | 5 (1.3) |
| **Other sarcoma** | 12 (3.0) |
| **Haemophagocytic lymphohistiocytosis** | 1 (0.3) |
| **Epatoblastoma** | 4 (1.0) |
| **Other solid tumor** | 5 (1.3) |

**Supplementary Table S2.** Rates of colonization and infection by resistant bacteria

|  | **% (n/tot)** |
| --- | --- |
| ***E. Coli*** |  |
|  | 22.8% (21/92) BSI with previous intestinal colonization (11 ESBL, 4 CPE, 1 ESBL+CPE, 5 other type) |
|  | 19/92 (20.7) ESBL+ BSI |
|  | ESBL+ in 6/21 (28.6) with previous colonization (all 6 with ESBL) |
|  | ESBL+ in 13/71 (18.3) without previous colonization |
|  | 1/92 (1.1) Carbapenemasi + (without previous colonization) |
| ***K. Pneumoniae*** |  |
|  | 13/49 (26.5) BSI with previous intestinal colonization (3 ESBL, 9 CPE, 1 ESBL+CPE) |
|  | 20/49 (40.8) ESBL+ BSI |
|  | ESBL+ in 3/13 (23.1) with previous colon (2 with ESBL, 1 with CPE) |
|  | ESBL+ in 17/36 (47.2) without previous colonization |
|  | 8/49 (16.3) carbapenemase+ BSI |
|  | carbapenemase + in 7/13 (53.8) with previous colonization (1 with ESBL 5 with CPE, 1 ESBL+CPE) |
|  | carbapenemase + in 1/36 (2.8) without previous colonization |
| ***Enterobacter spp*** |  |
|  | 3/30 (10.0) BSI with previous intestinal colonization (1 ESBL, 2 CPE) |
|  | 4/30 (13.3) ESBL+ BSI |
|  | ESBL+ in 1/3 (33.3) with previous colonization (1 ESBL) |
|  | ESBL+ in 3/27 (11.1) without previous colonization |
|  | 4/30 (13.3) carbapenemase+ |
|  | carbapenemase + in 2/3 (66.7) with previous colonization (2 CPE) |
|  | Carbapenemase+ in 2/27 (7.4) without previous colonization |
| ***Pseudomonas aeruginosa*** | 9/48 (18.8) intestinal colonization (7 ESBL, 2 other type) |
|  | 0 ESBL+ BSI |
|  | 2/48 (4.2) carbapenemase+ |
|  | 0/9 with previous colonization |
|  | 2/38 (5.1) without previous colonization |

**Supplementary Table S3.** Rates of microbiological appropriateness of empiric antibiotic therapy according to treatment phase

|  | **BSI episodes (n = 510)** | **First diagnosis**  **(N=311)** | **Relapse and auto HCT**  **(N=96)** | **Allo HCT**  **(N=103)** |
| --- | --- | --- | --- | --- |
| **Microbiological appropriateness** | 418 (82.0) | 250 (80.4) | 82 (85.4) | 86 (83.5) |
| No | 58 (11.4) | 36 (11.6) | 9 (9.4) | 13 (12.6) |
| Missing | 34 (6.7) | 25 (8.0) | 5 (5.2) | 4 (3.9) |
| **Microbiological appropriateness** (excluding missing data) | 418/476 (87.8) | 250/286 (87.4) | 82/91 (90.1) | 86/99 (86.9) |

**Supplementary Table S4.** 30- and 90-days outcome and clinical severity of BSI episode according to etiology, treatment phase and presence of MDR isolate

|  | **Etiology** | | | | | **Treatment phase** | | | **MDR – for gram neg only** | | | **Total** |
| --- | --- | --- | --- | --- | --- | --- | --- | --- | --- | --- | --- | --- |
|  | **Gram**  **pos** | **Gram**  **neg** | **Mixed** | **Fungi** | **Mycobacteria** | **First**  **diagnosis** | **Relapse and auto HCT** | **Allo HCT** | **Missing** | **No MDR** | **MDR** |  |
| **30 days outcome** | **N=222** | **N=213** | **N=25** | **N=2** | **N=1** | **N=292** | **N=79** | **N=92** | **N=24** | **N=174** | **N=35** | **N=463**** |
| Complete resolution | 189 (85.1) | 185 (86.9) | 20 (80.0) | 1 (50.0) | 1 (100.0) | 269 (92.1) | 66 (83.5) | 61 (66.3) | 23 (95.8) | 148 (85.1) | 30 (85.7) | 396 (85.5) |
| Clinical improvement,  therapy still ongoing | 13 (5.9) | 11 (5.2) | 3 (12.0) | 0 (0.0) | 0 (0.0) | 15 (5.1) | 6 (7.6) | 6 (6.5) | 1 (4.2) | 10 (5.7) | 2 (5.7) | 27 (5.8) |
| Stable | 1 (0.5) | 3 (1.4) | 0 (0.0) | 0 (0.0) | 0 (0.0) | 0 (0.0) | 2 (2.5) | 2 (2.2) | 0 (0.0) | 3 (1.7) | 0 (0.0) | 4 (0.9) |
| Clinical worsening | 6 (2.7) | 2 (0.9) | 1 (4.0) | 0 (0.0) | 0 (0.0) | 4 (1.4) | 3 (3.8) | 2 (2.2) | 0 (0.0) | 2 (1.1) | 1 (2.9) | 9 (1.9) |
| *Missing data* | 13 (5.9) | 12 (5.6) | 1 (4.0) | 1 (50.0) | 0 (0.0) | 4 (1.4) | 2 (2.5) | 21 (22.8) | 0 (0.0) | 11 (6.3) | 2 (5.7) | 27 ** (5.8) |
| **90 days outcome** | **N=186** | **N=185** | **N=20** | **N=1** | **N=1** | **N=249** | **N=62** | **N=82** | **N=19** | **N=155** | **N=27** | **N=393***** |
| Complete resolution | 171 (91.9) | 169 (91.4) | 19 (95.0) | 1 (100.0) | 1 (100.0) | 241 (96.8) | 58 (93.5) | 62 (75.6) | 19 (100.0) | 141 (91.0) | 24 (88.9) | 361 (91.9) |
| Clinical improvement,  therapy still ongoing | 3 (1.6) | 1 (0.5) | 1 (5.0) | 0 (0.0) | 0 (0.0) | 4 (1.6) | 1 (1.6) | 0 (0.0) | 0 (0.0) | 2 (1.3) | 0 (0.0) | 5 (1.3) |
| Stable | 1 (0.5) | 2 (1.1) | 0 (0.0) | 0 (0.0) | 0 (0.0) | 1 (0.4) | 1 (1.6) | 1 (1.2) | 0 (0.0) | 1 (0.6) | 1 (3.7) | 3 (0.8) |
| Clinical worsening | 2 (1.1) | 1 (0.5) | 0 (0.0) | 0 (0.0) | 0 (0.0) | 1 (0.4) | 0 (0.0) | 2 (2.4) | 0 (0.0) | 1 (0.6) | 0 (0.0) | 3 (0.8) |
| *Missing data* | 9 (4.8) | 12 (6.5) | 0 (0.0) | 0 (0.0) | 0 (0.0) | 2 (0.8) | 2 (3.2) | 17 (20.7) | 0 (0.0) | 10 (6.5) | 2 (7.4) | 21** (5.3) |
| **Clinical severuty of infection** | **N=239** | **N=242** | **N=25** | **N=1** | **N=3** | **N=311** | **N=96** | **N=103** | **N=29** | **N=191** | **N=42** | N=510 |
| Severe infection**** | 57 (23.8) | 81 (33.5) | 7 (28.0) | 0 (0.0) | 1 (33.3) | 63 (20.3) | 34 (35.4) | 49 (47.6) | 12 (41.4) | 61 (31.9) | 14 (33.3) | 146 (28.6) |
| Death due to infection | 6 (2.5) | 13 (5.4) | 1 (4.0) | 0 (0.0) | 1 (33.3) | 6 (1.9) | 7 (7.3) | 8 (7.8) | 2 (6.9) | 9 (4.7) | 3 (7.1) | 21 (4.1) |
| PICU admission | 10 (4.2) | 23 (9.5) | 4 (16.0) | 0 (0.0) | 1 (33.3) | 20 (6.4) | 6 (6.3) | 12 (11.7) | 4 (13.8) | 17 (8.9) | 5 (11.9) | 38 (7.5) |
| Respiratory support | 9 (3.8) | 21 (8.7) | 3 (12.0) | 0 (0.0) | 1 (33.3) | 17 (5.5) | 5 (5.2) | 12 (11.7) | 6 (20.7) | 11 (5.8) | 6 (14.3) | 34 (6.7) |
| Fluid support | 53 (22.2) | 66 (27.3) | 6 (24.0) | 0 (0.0) | 1 (33.3) | 53 (17.0) | 28 (29.2) | 45 (43.7) | 8 (27.6) | 52 (27.2) | 11 (26.2) | 126 (24.7) |
| Vasopressor support | 11 (4.6) | 26 (10.7) |  | 0 (0.0) | 5 (20.0) | 20 (6.4) | 5 (5.2) | 18 (17.5) | 5 (17.2) | 21 (11.0) | 4 (9.5) | 43 (8.4) |
| Renal support | 4 (1.7) | 7 (2.9) |  | 0 (0.0) | 1 (4.0) | 7 (2.3) | 1 (1.0) | 5 (4.9) | 3 (10.3) | 2 (1.0) | 2 (4.8) | 13 (2.5) |

** 47 cases excluded: dead before 30 days

*** 117 cases excluded: dead before 90 days

**** composite outcome of severe infection: at least 1 among PICU admission, respiratory support, fluid support, amminic support, renal support or death due to infection

**Supplementary Table S5.** Clinical characteristics of 21 patients deceased due to infectious episode

| **GP/GN** | **Pathogen** | **Diagnosis** | **Treatment phase** | **PICU admiss.** | **Respiratory supp.** | **Fluid supp.** | **Renal supp.** | **Vasopressor supp.** | **T > 39°C** | **Hypoxemia** | **Hypothension** | **ANC <100** |
| --- | --- | --- | --- | --- | --- | --- | --- | --- | --- | --- | --- | --- |
| GN | *P. Aeruginosa* | Non malign. | Allo HCT | Yes | Yes | Yes | No | Yes | No | Yes | . | Yes |
| GN | *K. Pneumoniae* | ALL | Allo HCT | No | Yes | Yes | No | No | Yes | Yes | Yes | Yes |
| GN | *Acinetobacter baumannii* | AML | Relapse or Auto HCT | No | No | No | No | No | Yes | . | . | No |
| GN | *Klebsiella oxytoca* | NHL | 1st diagn. | Yes | No | Yes | No | No | Yes | . | . | No |
| GP | *S. Epidermidis* | AML | 1st diagn. | No | No | No | No | No | Yes | . | . | Yes |
| GP | *Enterococcus faecium* | AML | Relapse or Auto HCT | No | Yes | Yes | No | No | No | . | . | Yes |
| GN | *Enterobacter cloacae* | ALL | Relapse or Auto HCT | No | No | Yes | No | No | No | . | . | Yes |
| GN | *K. pneumoniae* | ALL | 1st diagn. | Yes | Yes | No | No | Yes | No | . | . | Yes |
| GN | *Aeromonas sobria* | ALL | 1st diagn. | Yes | Yes | Yes | Yes | Yes | No | Yes | Yes | Yes |
| Mix. | *E.cloacae+Staph. haem.+Staph.epid.* | ALL | Relapse or Auto HCT | Yes | No | Yes | No | Yes | No | . | Yes | Yes |
| GP | *Enterococcus faecium* | ALL | Allo HCT | Yes | Yes | Yes | Yes | No | No | . | . | No |
| GN | *P. aeruginosa* | ALL | Relapse or Auto HCT | No | No | Yes | No | No | No | Yes | . | Yes |
| Fung | *Candida glabrata* | AML | 1st diagn. | Yes | Yes | Yes | Yes | Yes | Yes | Yes | Yes | Yes |
| GN | *E.coli+P. aeruginosa* | AML | Allo HCT | Yes | Yes | Yes | No | Yes | Yes | Yes | Yes | Yes |
| GN | *Enterobacter aerogenes* | ALL | Relapse or Auto HCT | Yes | Yes | Yes | No | Yes | No | Yes | . | Yes |
| GN | *E. coli* | ALL | Relapse or Auto HCT | No | No | No | No | No | No | . | Yes | No |
| GP | *Streptococcus mitis/oralis* | ALL | Allo HCT | Yes | No | Yes | Yes | Yes | Yes | . | Yes | Yes |
| GP | *Clostridium septicum* | ALL | 1st diagn. | Yes | Yes | Yes | Yes | Yes | No | Yes | Yes | Yes |
| GN | *E. coli* | SAA | Allo HCT | Yes | Yes | Yes | Yes | Yes | No | Yes | . | Yes |
| GP | *Enterococcus faecium* | SAA | Allo HCT | Yes | Yes | Yes | No | Yes | No | . | . | No |
| GN | *K. pneumoniae* | ALL | Allo HCT | Yes | Yes | Yes | Yes | Yes | No | . | . | Yes |

**Supplementary Table S5.** Infection related, 30-day, 90-day and overall mortality of BSI episodes according to MDR status, etiology and treatment phase

|  | **BSI episodes** | **Death** | **Infection related mortality** | **Death** | **30-days mortality CI – n (IC)** | **Death** | **90-days mortality CI – n (IC)** | **Death** | **Overall survival** |
| --- | --- | --- | --- | --- | --- | --- | --- | --- | --- |
| **MDR GN BSI** | 175 | 3 | 7.14  (1.81-17.61) | 5 | 5.75  (2.93-9.90) | 5 | 5.75  (2.93-9.90) | 6 | 9.26  (5.39-14.38) |
| No MDR GN BSI | 42 | 8 | 4.59  (2.15-8.44) | 10 | 12.03  (4.33- 23.96) | 10 | 12.03  (4.33- 23.96) | 15 | 16.03  (6.14-30.08) |
| P |  |  | 0.5 |  | 0.2 |  | 0.2 |  | 0.2 |
| **GP** | 239 | 6 | 2.73  (1.12-5.59) | 8 | 3.36  (1.58-6.23) | 14 | 6.15  (3.52-9.81) | 28 | 13.52  (9.24-18.61) |
| **GN** | 242 | 13 | 5.40  (3.02-8.77) | 19 | 7.90  (4.93-11.76) | 19 | 7.90  (4.93-11.76) | 27 | 12.21  (8.26-16.99) |
| **Mixed** | 25 | 1 | 5.00  (0.31-21.07) | 0 | 0.0 | 2 | 8.73  (1.42-24.73) | 3 | 13.29  (3.18-30.71) |
| **Fungi** | 3 | 1 | - | 0 | - | 1 | - | 1 | - |
| **Mycobacteria** | 1 | 0 | - | 0 | - | 0 | - | 0 | - |
|  |  |  | 0.1 |  | 0.03 |  | 0.4* |  | 1 |
| **First diagnosis** | 311 | 6 | 2.09  (0.86-4.29) | 7 | 2.25  (1.00-4.39) | 11 | 3.75  (1.98-6.39) | 21 | 7.82  (4.98-11.46) |
| **Relapse and auto HCT** | 96 | 7 | 7.80  (3.37-14.66) | 10 | 10.69  (5.44-17.95) | 12 | 13.24  (6.59-21.16) | 20 | 25.23  (16.00-35.53) |
| **Allo HCT** | 103 | 8 | 7.87  (3.66-14.18) | 10 | 9.71  (4.95-16.38) | 13 | 12.72  (7.12-20.00) | 18 | 18.33  (11.35-26.64) |
|  |  |  | **0.007** |  | 0.0006 |  | 0.0005 |  | <0.0001 |

* Gram positive vs gram negative
